# Combined genomic and transcriptomic analysis reveals the contribution of tandem duplication genes to low-temperature adaptation in perennial ryegrass

**DOI:** 10.3389/fpls.2023.1216048

**Published:** 2023-07-12

**Authors:** Wei Wang, Xiaoning Li, Shugao Fan, Yang He, Meng Wei, Jiayi Wang, Yanling Yin, Yanfeng Liu

**Affiliations:** ^1^School of Resources and Environmental Engineering, Ludong University, Yantai, China; ^2^The Engineering Research Institute of Agriculture and Forestry, Ludong University, Yantai, China

**Keywords:** comparative analysis, abiotic stress, low-temperature adaptation, tandem duplication genes, perennial ryegrass

## Abstract

Perennial ryegrass (*Lolium perenne* L.) is an agronomically important cool-season grass species that is widely used as forage for ruminant animal production and cultivated in temperate regions for the establishment of lawns. However, the underlying genetic mechanism of the response of *L. perenne* to low temperature is still unclear. In the present study, we performed a comprehensive study and identified 3,770 tandem duplication genes (TDGs) in *L. perenne*, and evolutionary analysis revealed that *L. perenne* might have undergone a duplication event approximately 7.69 Mya. GO and KEGG pathway functional analyses revealed that these TDGs were mainly enriched in photosynthesis, hormone-mediated signaling pathways and responses to various stresses, suggesting that TDGs contribute to the environmental adaptability of *L. perenne*. In addition, the expression profile analysis revealed that the expression levels of TDGs were highly conserved and significantly lower than those of all genes in different tissues, while the frequency of differentially expressed genes (DEGs) from TDGs was much higher than that of DEGs from all genes in response to low-temperature stress. Finally, in-depth analysis of the important and expanded gene family indicated that the members of the ELIP subfamily could rapidly respond to low temperature and persistently maintain higher expression levels during all low temperature stress time points, suggesting that ELIPs most likely mediate low temperature responses and help to facilitate adaptation to low temperature in *L. perenne*. Our results provide evidence for the genetic underpinning of low-temperature adaptation and valuable resources for practical application and genetic improvement for stress resistance in *L. perenne*.

## 1 Introduction

As one of the major environmental factors, low temperature can severely inhibit plant growth, development and productivity, and is also considered to be a principal determinant of biodiversity geographic distribution patterns (Humphreys and Linder, 2013). Low temperature can result in a variety of unfavorable changes in plant physiological processes by directly inhibiting metabolic reactions and indirectly causing osmotic and oxidative stresses (Diao et al., 2020). In response to this adverse environmental factor, plants successfully evolved a set of sophisticated mechanisms that allow them to withstand freezing (< 0°C) or chilling stress (0–15°C) (Ding et al., 2020). Therefore, unraveling the low temperature-adapted molecular mechanisms of plants may provide interesting targets for developing and selecting low temperature-tolerant genotypes using breeding or genomic approaches, which seems particularly important in high-latitude areas and high-altitude areas. Over the past two decades, much progress has been made in identifying the crucial components (e.g. messenger molecules, protein kinases, phosphatases and transcription factors) involved in low-temperature tolerance and dissecting their regulatory mechanisms (Ding et al., 2019). Accumulating evidence indicates that plants perceive cold signals at different sensory levels, including cell membrane fluidity hypothesis, calcium channels and phytochrome. After sensing the cold signals, the signals are transduced by second messengers (e.g. calcium, reactive oxygen species and nitric oxide). Following the transduction of the cold signals into the nucleus, the cold signaling pathways, including CBF-dependent and CBF-independent pathway, are activated and the expression levels of many genes are altered to mediate the low-temperature tolerance in plants (Ding et al., 2019; Zhang et al., 2020; Ding and Yang, 2022). The C-repeat/DREB binding factors (CBFs) have been identified as an important transcription factor that regulate the expression of low-temperature-responsive genes, and overexpressing *CBF1* in *Arabidopsis thaliana* increased the expression of *COR* genes and enhanced freezing tolerance (Jaglo-Ottosen et al., 1998; Shi et al., 2018). Low-temperature stress responses are triggered to increase plant survival, but they generally sacrifice plant growth by repressing cell division and expansion (Zhang et al., 2020; Ding and Yang, 2022). Increasing evidence indicates that this low-temperature stress-specific sacrifice-for-survival mechanism is due to limit in energy/carbon supply which mainly results from the active suppression of growth by stress signaling pathways (Zhang et al., 2020). For example, jasmonate signaling, auxin signaling and other stress signaling pathways were identified in Antarctic moss *Pohlia nutans* and founded that these pathways might contribute to *P. nutans* acclimating to cold stress (Liu et al., 2022c).

Perennial ryegrass (*Lolium perenne* L.) is a wild perennial grass belonging to the family Pooideae, subfamily Pooideae, tribe Lolieae and is considered to be an important and widespread cool-season grass species (Förster et al., 2018). It is widely used as a forage species for ruminant animal production in temperate regions and as an alternative and renewable bioenergy source and is also widely cultivated for the establishment of lawns in urban areas (Dąbrowski et al., 2023). Pooideae, as one of the most species-rich grass subfamilies, occupy the coldest climate space, suggesting that they have successfully adapted to and diversified in cool climate ecosystems (Vigeland et al., 2013). Increasing evidence has inferred adaptation to cooler environments at the base of the Pooideae phylogeny (Edwards and Smith, 2010), and five gene families, including C-repeat-binding factors (CBF), dehydrins (DHN), chloroplast-targeted cold-regulated proteins (ctCOR), ice recrystallization inhibition proteins (IRIP) and fructosyl transferases (FST), may have important functions in response to cold stress and acclimation in core Pooideae (Schubert et al., 2019). As a member of the core Pooideae species, the understanding of its low-temperature tolerance of perennial ryegrass is still mainly focused on the physiological and molecular mechanisms, their underlying genetic basis of adaptation to low temperature at the whole genome level needs further exploration.

Gene duplication is an important evolutionary mechanism and is considered a major driving force for expanding the functionality of a multigene family and providing new genes for evolutionary novelty and ecological adaptation (Zhou et al., 2019). Whole-genome duplication (WGD), tandem duplication, duplication mediated by transposable elements, segmental duplication and retroduplication are proposed as the main mechanisms for gene duplication (Panchy et al., 2016). Among them, tandem duplication resulting from unequal crossing over is a prevalent phenomenon that occurs multiple times in all angiosperms and plays significant roles in conferring plant adaptation to changing environments (Yu et al., 2015; Das Laha et al., 2020). For example, tandem duplication events contributed to eudicot adaptation during paleoenvironmental changes (Guo et al., 2022), were involved in tolerance to salt stress in poplar (Ma et al., 2013), and 27% of tandem element-mediated duplicates were responsive to abiotic stress in Arabidopsis (Wu et al., 2012). Research about the influence of tandem duplication on duplicate retention indicated that those genes involved in stress responses generally have an elevated probability of retention following tandem duplication, and new tandem gene paralogs are continuously generated with the occurrence of duplication events, likely providing a pool of high dynamic targets for adaptive evolution to rapidly changing environments (Hanada et al., 2008; Zhong et al., 2018). For example, gene collinearity and phylogeny analyses uncovered that the C-repeat binding factors/dehydration-responsive element binding protein 1 (*CBF/DREB1*) is an innovation resulted from tandem duplication-derived DREB III gene, and subsequent ϵ-whole genome duplication led to Clades I and II of *CBF/DREB1* in ancient angiosperms. Among them, Clades I and their parent DREB III genes showed cold-insensitivity, while Clade II genes evolved into cold-sensitive response and underwent independent expansions by convergent evolution (conserved in cold induction) in eudicots and monocots, suggesting that the duplicated *CBF/DREB1* genes mediated the rewiring of CBFs/DREB1s-regulatory network for cold tolerance (Nie et al., 2022). Similarly, a tandem array of *CBF/DREB1* genes located in a major freezing tolerance QTL region were identified on *Medicago truncatula* chromosome 6 (Tayeh et al., 2013), and the expanded gene families (e.g. *CBF* and *LEA*) might drive Pooideae grasses from tropical to temperate regions (Zhong et al., 2018). Moreover, expansion of the early light-induced proteins (ELIPs) was previously reported in some plant species, including *Boea hydrometrica* (Xiao et al., 2015), *Selaginella lepidophylla* (VanBuren et al., 2018b), *Lindernia subracemosa* (VanBuren et al., 2018a) and biocrust moss *Syntrichia caninervis* (Silva et al., 2021). As the subfamily of the light-harvesting chlorophyll a/b-binding protein (Lhc) superfamily, *ELIPs* encode proteins act as photoprotectants by binding to chlorphylls and carotenoids to protect them against photooxidative damage involved in high light stress (Silva et al., 2021), as well as participate in response to desiccation, cold and drought stresses (Adamska and Kloppstech, 1994; VanBuren et al., 2019). For example, overexpression of a *M. truncatula ELIP* in *Nicotiana benthamiana* increased resistance to freezing and chilling, and overexpression of a *Craterostigma plantagineum ELIP* in *M. truncatula* increased drought tolerance (Araújo et al., 2013). Recently, comparative genomic and transcriptomic analyses uncovered that ELIPs expanded in resurrection plants through tandem gene duplication and the increased abundance of ELIPs help facilitated the rapid recovery for most resurrection plants under desiccation and rehydration conditions (VanBuren et al., 2019). In addition, tandem duplication events also play important roles in plant growth, development and metabolic processes (Hofberger et al., 2013; Xu et al., 2020b). Unfortunately, tandem duplication genes (TDGs) and their possible contributions to the genetic basis of low-temperature adaptation in *L. perenne* are still ambiguous.

To better illustrate the molecular evolutionary mechanisms of TDGs underlying low-temperature stress in *L. perenne*, we performed a comprehensive study to identify the TDG signatures in the *L. perenne* genome and analyze their evolutionary contributions. Subsequently, GO and KEGG enrichment analyses were performed to investigate the functions of the TDGs specific for *L. perenne* and Pooideae lineage species. Moreover, the expression patterns of the TDGs in different tissues and their response to low-temperature stress were analyzed. Finally, the potential and important gene family involved in adaptation to low-temperature stress in *L. perenne* was also investigated. The information generated in this study facilitates the understanding of low-temperature adaptation and provides valuable genetic resources for further studies on low-temperature-related traits in *L. perenne*.

## 2 Materials and methods

### 2.1 Genomic datasets

A total of five sequenced Poaceae genomes, including four Pooideae genomes of *L. perenne* (Lolium_2.6.1_V3), *Hordeum vulgare* (HvulgareMorex_702_V3), *Brachypodium distachyon* (Bdistachyon_556_v3.2) and *Achnatherum splendens* (Unlabeled) and one Oryzoideae genome of *Oryza sativa* (Osativa_323_v7.0), were subjected to comparative genomic analysis. The protein sequences and General Feature Formant (GFF) files of all studied species were downloaded from Phytozome database (version 13) (https://phytozome-next.jgi.doe.gov/) (Goodstein et al., 2012), except *A. splendens* was downloaded from the National Genomics Data Center (https://bigd.big.ac.cn/?lang=en) using the accession of PRJCA00214.

### 2.2 TDG and evolution analysis

The longest translation form of the protein-coding genes from five Poaceae species was selected to represent each gene, and then all filtered protein sequences of each genome were subjected to an all-against-all BLASTP (version 2.7.1+) with an E-value < 1e-10 and max_target_seqs set as 10 to search for potential homologous gene pairs (Altschul et al., 1997). Then, the blast results and GFF file of each species were analyzed using MCScanX software to identify the tandem duplicated gene pairs with the following parameter settings: the alignment significance (E_VALUE) set as 1e-05, the final score (MATCH_SCORE) set as 50, the number of genes required to call a collinear block (MATCH_SIZE) set as 5 and the maximum gaps (MAX_GAPS) set as 25 (Wang et al., 2012). Subsequently, those genes falling in the identified collinear blocks with closely adjacent homologous gene (no more than one gene separating them) were defined as tandem duplication genes according to the identification standards in MCScanX (Wang et al., 2012). To estimate the duplication events of TDGs, the nonsynonymous (Ka) and synonymous substitution (Ks) frequencies of each duplicated gene pair were calculated by PAML (version 4.9 h) using the yn00 program and YN model (Yang, 1997). Subsequently, the peak Ks was used to estimate the approximate dates of duplication events following the Formula *T* = Ks/2λ by using an average substitution rate of 6.5e-9 for grasses (Gaut et al., 1996). Finally, the Ka/Ks ratio was also calculated to evaluate the selection pressure for each of the duplicated gene pairs (Androsiuk et al., 2022).

### 2.3 GO term and KEGG pathway enrichment analysis

To maintain comparability among different species, the protein sequences of each species were subjected to functional annotation by eggNOG-mapper (version 2.1.9) with default parameters (Cantalapiedra et al., 2021). Then, Gene Ontology (GO) term and Kyoto Encyclopedia of Genes and Genomes (KEGG) pathway functional enrichment analyses for TDGs of each species were performed using the clusterProfiler package in R software (version 4.2.2) (Wu et al., 2021), with all the protein-coding genes of each species as the background gene set. Finally, the functional enrichment results were visualized by the ggplot2 package in R software (version 4.2.2) (Wu et al., 2021).

### 2.4 Expression analysis

To investigate the in silico expression profiles of the *L. perenne* TDGs in different tissues, the RNA-Seq data from six tissues, including leaf sheath, inflorescence, mature leaf, meristem, root and stem, which were collected from the perennial ryegrass genotype P226/135/16, were downloaded from the National Center for Biotechnology Information (NCBI) databases (BioProject accession: PRJNA222646) (Farrell et al., 2014). The raw reads were trimmed using Trimmomatic (version 0.36) (Bolger et al., 2014), and then the obtained clean reads were aligned to the reference genome using HISAT2 (version 2.1.0) (Kim et al., 2015). The FPKM (fragments per kilobase per million mapped reads) value of individual genes was estimated by StringTie software (version 2.2.1) (Pertea et al., 2016). The log2(FPKM+1) values of the TDGs and all genes were used to compare the expression patterns in different tissues, and the results were visualized by the pheatmap and ggplot2 packages in R software (version 4.2.2) (Wu et al., 2021).

To examine genome-wide responses to cold stress, the RNA-Seq data (three biological replicates) from a low temperature-adapted ecotype Falster, which was subjected to low temperature stress and sampled at 0, 9, 13 and 17 d, were downloaded from ArrayExpress with the accession number E-MTAB-2779 (Abeynayake et al., 2015). Trimmomatic (version 0.36) (Bolger et al., 2014), HISAT2 (version 2.1.0) (Kim et al., 2015) and StringTie (version 2.2.1) (Pertea et al., 2016) software were also used to process the RNA-Seq reads. Then, differential expression analysis for the different sample comparisons was performed using the R package DESeq2 with a false discovery rate (FDR) < 0.05 and |log2 (FoldChange)| ≥ 1 as the threshold to identify the differentially expressed genes (DEGs) (Love et al., 2014).

### 2.5 Identification and analysis of the light-harvesting chlorophyll a/b-binding superfamily

To identify the putative light-harvesting chlorophyll a/b-binding (Lhc) superfamily genes in *L. perenne*, the protein sequences of 34 *AtLhc* genes were collected from the Phytozome database (version 13) (https://phytozome-next.jgi.doe.gov/) (Goodstein et al., 2012). Then, a local protein database was constructed by the BLAST tool (version 2.7.1+), and a BLASTP search was performed using the 34 known AtLhc protein sequences with an e-value of 1e-10, keeping the putative protein sequences with lengths greater than 100 amino acids. All candidate sequences were examined to confirm the presence of the conserved CB domain (PF00504) using MOTIF Search (https://www.genome.jp/tools/motif/) and SMART (http://smart.embl-heidelberg.de/) (Letunic et al., 2015). Finally, the molecular weight (Mw) and theoretical isoelectric point (pI) for each LpLhc protein were estimated by the ExPASy Compute pI/Mw tool (https://web.expasy.org/protparam/) (Wilkins et al., 1999). Moreover, to investigate the distribution patterns of ELIP subfamily genes in Poaceae, the members of the ELIP subfamily of another seven species, including three Pooideae genomes of *H. vulgare* (HvulgareMorex_702_V3), *B. distachyon* (Bdistachyon_556_v3.2) and *A. splendens* (Unlabeled), one Oryzoideae genome of *O. sativa* (Osativa_323_v7.0) and three Panicoideae genomes of *Zea mays* (Zmays_284_Ensembl-18), *Sorghum bicolor* (Sbicolor_454_v3.1.1) and *Setaria italica* (Sitalica_312_v2.2) were also identified using the same identification strategy.

## 3 Results

### 3.1 Identification and analyses of TDGs in perennial ryegrass and other grasses

**Figure 1:**
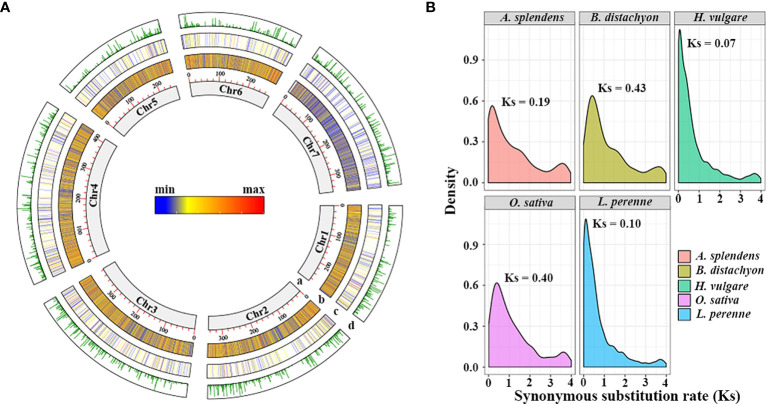
The landscape characteristics of the *L. perenne* genome and the distribution of synonymous substitution levels (Ks) between tandem duplication gene pairs. **(A)** Tracks from inside (a) to outside (d) correspond to (a) Chromosome size with units in Mb; (b) density of genes; (c) density of TDGs; (d) Ks of TDG pairs. **(B)** Distribution of Ks calculated by the TDG gene pairs among studied species.

The *L. perenne* genome sequence consists of 2,311 Mb of DNA and 70,534 protein-coding genes (Nagy et al., 2022). Using MCScanX software and the downstream analysis tool incorporated into the MCScanX package, a total of 3,770 TDGs (5.68% of the gene set) were identified in the *L. perenne* genome, with a lower frequency than other studied grass species (**Table S1**). Among them, 728 TDGs (19.31% of total TDGs) were located on chromosome 4 (Chr4), which had the highest number of TDGs, while Chr5 had the lowest number of TDGs (396 TDGs, 10.50% of total TDGs) (**Figure 1A**; **Table S2**). The synonymous substitution rates (Ks) of the TDG pairs were calculated for 2,042 gene pairs, and the distribution of Ks showed a single peak value at Ks = 0.10, suggesting that *L. perenne* might have undergone a duplication event at approximately 7.69 Mya (**Figure 1B**). In addition, the selection pressure acting on TDG pairs was inferred from the ratio of nonsynonymous (Ka) to synonymous (Ks) substitution values (Ka/Ks) (**Table S3**), our results showed that 1,842 TDG pairs (90.21% of total TDG pairs) had Ka/Ks values less than 1, whereas only 200 TDG pairs (9.79% of total TDG pairs) had Ka/Ks values greater than 1, indicating that most TDGs experienced strong purifying selection and that a small number underwent positive selection during the course of evolution.

Finally, statistical results showed that the number of TDGs in the same tandem cluster ranged from two to six, and the functional characteristics of TDGs with more than five genes in the same cluster were annotated. Our results indicated that most of these large-scale TDG clusters were involved in the stress response, including the F-box domain, glycoside hydrolase family, short-chain dehydrogenase/reductase SDR, AP2/ERF domain and BTB/POZ domain (**Table S4**).

### 3.2 The TDGs contributed to the environmental adaptability of *L. perenne*

**Figure 2:**
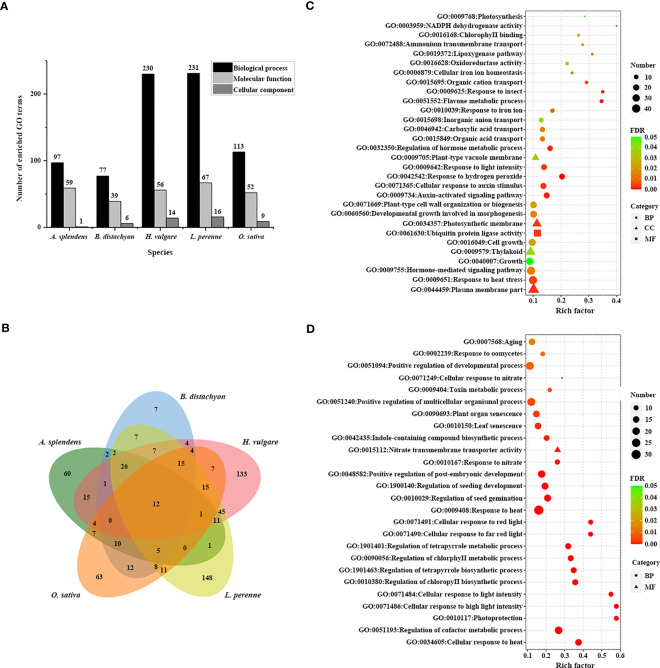
GO enrichment analysis of TDGs. **(A)** The number of enriched GO terms in each studied species. **(B)** Venn diagram of the number of shared and unique enriched GO terms among five species. **(C)** 30 unique enriched GO terms of TDGs in *L. perenne* compared with other analyzed species. **(D)** Pooideae-specific enriched GO terms compared with rice.

To reveal the genetic basis underlying the adaptation to the environment, we assessed the functions of 3,770 TDGs in *L. perenne*. Gene Ontology (GO) enrichment analysis indicated that these TDGs were significantly enriched in 314 GO terms (**Figures 2A, B**; **Table S5**). To investigate *L. perenne*-specific GO terms, we performed a comparative analysis of four grass species, including *O. sativa*, *B. distachyon*, *H. vulgare* and *A. splendens*, with *L. perenne*. The results revealed that the TDGs in *L. perenne* were enriched in 148 unique GO terms compared with those in the other analyzed species (**Figure 2C**; **Table S6**). These unique GO terms included hormone-mediated signaling pathway (GO:0009755), photosynthesis (GO:0009768), oxidoreductase activity (GO:0016628) and NADPH dehydrogenase activity (GO:0003959), which might be associated with adaptation to low temperature climates.

Pooideae, as they have successfully adapted to and diversified in cool climate ecosystems, are considered to be a cold-adapted lineage in Poaceae. To understand the low-temperature adaptation of Pooideae, the Pooideae-specific GO terms shared in *L. perenne*, *H. vulgare*, *B. distachyon* and *A. splendens* but absent in *O. sativa* were identified (**Figure 2D**; **Table S7**). Comparative analysis revealed that the TDGs in the four species shared enrichment in 26 Pooideae-specific GO terms, including photoprotection (GO:0010117) and cellular response to light intensity (GO:0071486), compared with the *O. sativa* genome, which are related to climate adaptation.

**Figure 3:**
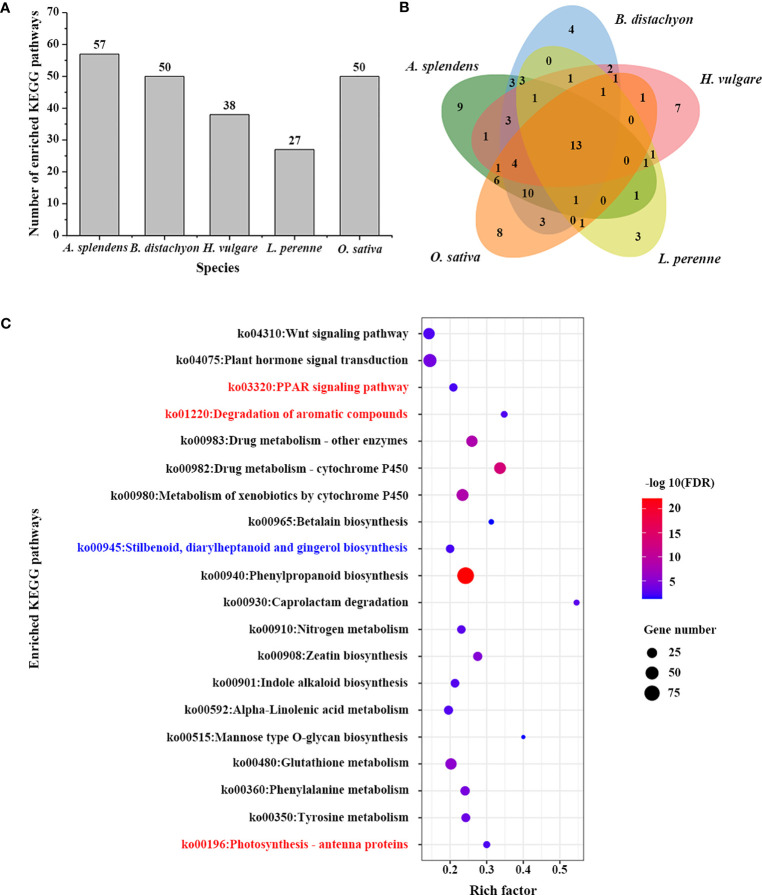
KEGG enrichment analysis of TDGs. **(A)** The number of enriched KEGG pathways in each studied species. **(B)** Venn diagram of the number of shared and unique enriched KEGG pathways among five species. **(C)** Enriched KEGG pathways of TDGs in *L. perenne*. The pathways in red are *L. perenne*-specific KEGG pathways compared with other four species. The pathway in blue is Pooideae-specific KEGG pathway compared with rice.

To further understand the complex biological functions of genes, we also performed a KEGG enrichment analysis to retrieve the enrichment pathways involved in those TDGs. A total of 27 significantly enriched pathways were identified for 3,770 TDGs from *L. perenne* (**Figures 3A, B**; **Table S8**). These enriched pathways included plant hormone signal transduction (ko04075), phenylpropanoid biosynthesis (ko00940) and metabolism of xenobiotics by cytochrome P450 (ko00980). In comparison with the other four studied species, 3 unique KEGG pathways, including photosynthesis - antenna proteins (ko00196), peroxisome proliferator-activated receptor (PPAR) signaling pathway (ko03320) and degradation of aromatic compounds (ko01220), were identified for *L. perenne* (**Figure 3C**). In addition, stilbenoid, diarylheptanoid and gingerol biosynthesis (ko00945) was the Pooideae-specific enrichment pathway compared with the *O. sativa* genome.

### 3.3 TDGs contributed to low-temperature adaptation

**Figure 4:**
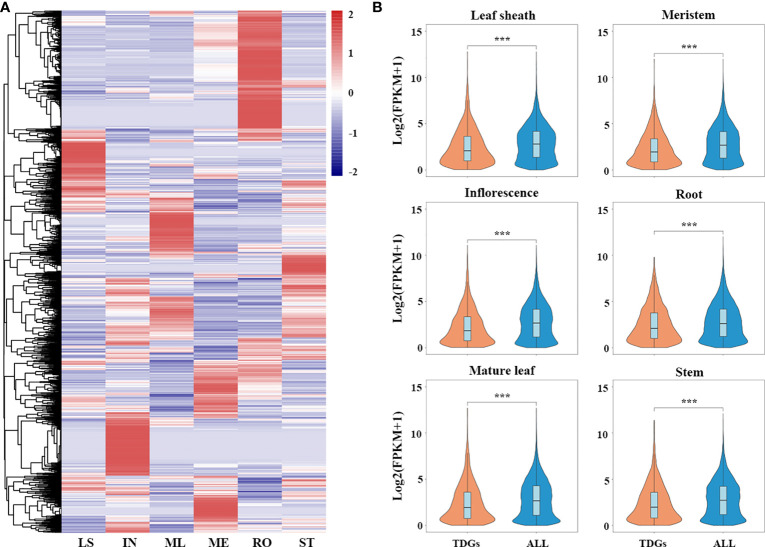
Transcriptomics of *L. perenne* in six different tissues. **(A)** Expression patterns of TDGs in six different tissues. The heatmap was generated from hierarchical cluster analysis of genes. LS, IN, ML, ME, RO and ST represent leaf sheath, inflorescence, mature leaf, meristem, root and stem, respectively. **(B)** Comparison of expression level between TDGs and all genes in six different tissues. *** indicate the differences between TDGs and all genes, ***p < 0.001.

To elucidate the spatial-temporal patterns of the TDGs in *L. perenne*, we reanalyzed the publicly available RNA-seq data (BioProject accession: PRJNA259941) of 18 samples in different tissues, including leaf sheaths, inflorescences, mature leaves, meristems, roots and stems (Farrell et al., 2014). The analysis results showed that most of the TDGs exhibited tissue-specific expression (**Figure 4A**). Among the 3,770 TDGs, 2,361 (62.6%) TDGs were expressed in at least one tissue. Gene enrichment analysis suggested that these 2,361 TDGs were enriched in a large number of stress-responsive GO functional categories, such as response to salt stress (GO:0009651), water deprivation (GO:0009414) and wounding (GO:0009611), and some GO terms were involved in plant development and adaptation to environmental stimuli, including regulation of hormone levels (GO:0010817), photoprotection (GO:0010117) and oxidoreductase activity (GO:0016684) (**Table S9**). In addition, we performed a comparative expression analysis between TDGs and all genes in *L. perenne*, and our results showed that the expression levels of TDGs were significantly lower than those of all genes in all six tissues (Wilcoxon rank-sum test, *P* < 2.2e - 16 in leaf sheaths, inflorescences, mature leaves, meristems and stems, and *P* = 1.2e - 12 in roots) (**Figure 4B**).

**Figure 5:**
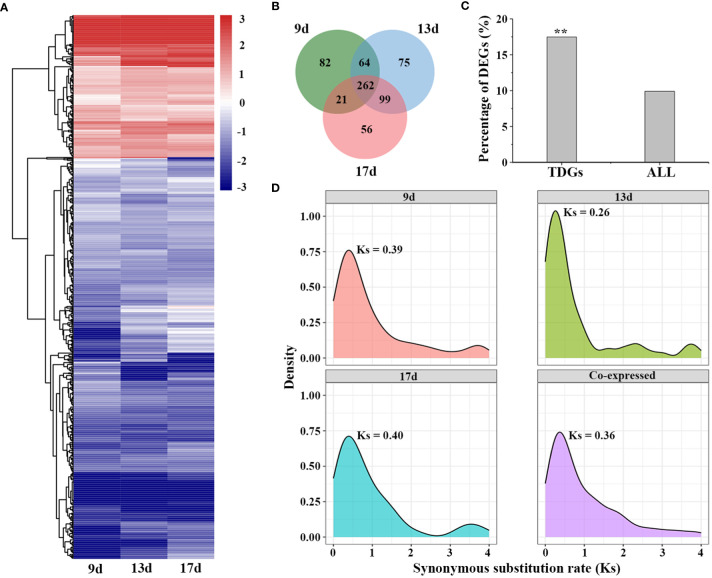
Expression patterns of TDGs under low temperature stress. **(A)** Expression of differentially expressed TDGs identified in leaves at each time point. The heatmap was generated from hierarchical cluster analysis of genes. **(B)** Venn diagram of the number of differentially expressed TDGs in leaves at each time point. **(C)** The percentage of DEGs derived from TDGs and from all genes under low temperature stress. **(D)** Distribution of Ks with different specific expression TDGs. ** indicate the differences between TDGs and all genes, **p < 0.01.

To further investigate the genetic mechanisms underlying low-temperature adaptation, we performed transcriptomic analysis under low-temperature stress in *L. perenne* using publicly available data (Abeynayake et al., 2015). Differentially expressed genes (DEGs) were identified under low temperature stress by comparing each time point (9, 13 and 17 d) with 0 d. A total of 429, 500 and 438 differentially expressed TDGs were identified in the 9, 13 and 17 d low temperature-stressed leaves, respectively (**Figures 5A, B**). Of the 3,770 TDGs, the expression levels of 659 (17.48%) TDGs were significantly altered by low-temperature stress for at least one time point (**Table S10**), and the frequency of DEGs derived from tandem duplication events was much higher than that of DEGs derived from all genes (6,582 out of 66,045 genes, 9.91%, χ^2^ test, *P* < 0.05) (**Figures 5B, C**). In addition, among the 659 differentially expressed TDGs, 262 DEGs (39.8%) were coexpressed in all samples (9, 13 and 17 d low temperature-stressed leaves), while 82 (12.4%), 75 (11.4%) and 56 (8.5%) DEGs were specifically expressed in 9, 13 and 17 d low temperature-stressed leaves, respectively (**Figure 5B**). Gene enrichment analysis suggested that 262 coexpressed TDGs were mainly enriched in some biological processes involved in stress responses, such as cellular response to abiotic stimulus (GO:0071214), cellular hormone metabolic process (GO:0034754) and photoprotection (GO:0010117), suggesting that these TDGs might participate in temperature sensing and most likely play a crucial role in the adaptation of *L. perenne* to low temperature (**Table S11**).

To further trace the evolutionary history of the differentially expressed TDGs that respond to low temperature stress, we recalculated the frequencies of synonymous substitution (Ks) for those TDGs that were specifically expressed in 9, 13 and 17 d low temperature-stressed leaves and coexpressed at three time points (**Figure 5D**). The distribution of Ks showed only a Ks peak specific for those coexpressed TDGs (Ks = 0.36), while two peaks including one peak between 0.26 (13 d) to 0.40 (17 d) and another peak between 3.55 (17 d) to 3.75 (9 d) for those specifically expressed TDGs, implying that the duplication events occurring approximately 20-31 Mya play an indispensable role for *L. perenne* to respond and adapt to low temperature stress.

### 3.4 The ELIP gene family possibly mediates low-temperature responses in *L. perenne*

**Figure 6:**
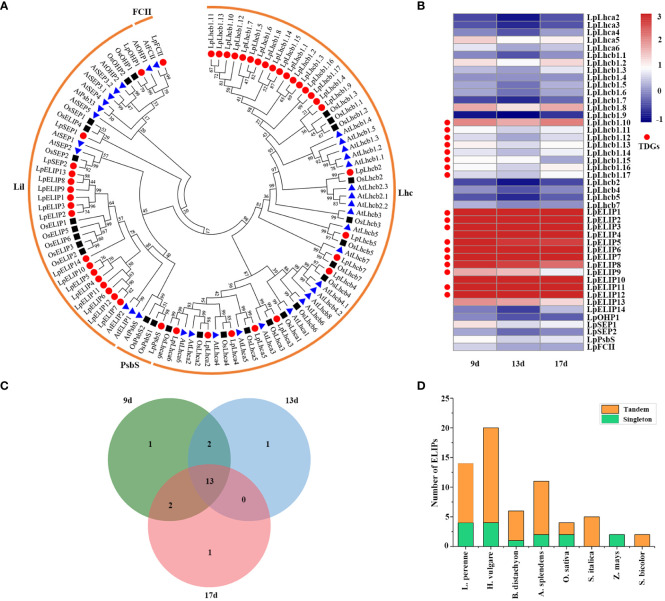
Analysis of the light-harvesting chlorophyll a/b-binding protein superfamily. **(A)** Phylogenetic tree of light-harvesting chlorophyll a/b-binding proteins from perennial ryegrass, Arabidopsis and rice. Bootstrap values in percentage (1000 replicates) are labeled on the nodes. Signs of different shapes represent Lhc proteins from perennial ryegrass (red round), rice (black square) and Arabidopsis (blue triangle). **(B)** Heat map of the expression profiles of the *Lhc* genes in perennial ryegrass under low temperature stress. The genes marked with red round represent TDGs. **(C)** Venn diagrams with numbers of differentially expressed *Lhcs* in perennial ryegrass under low temperature stress. **(D)** ELIP composition in eight sequenced Poaceae species. Tandemly duplicated ELIPs are plotted in orange, and single copy ELIPs are plotted in green.

GO and KEGG pathway enrichment analyses revealed that 3,770 TDGs were significantly enriched in photosynthesis or photoprotection functions. The enriched term photoprotection (GO:0010117) or Photosynthesis-antenna proteins pathway (ko00196) contains many light-harvesting chlorophyll a/b-binding proteins (**Tables S5**; **S8**), which play important roles in multiple processes, particularly roles in stress responses. To assess the function of these genes, we used BLASTP to search the *L. perenne* genome and identified the best-hit genes. A total of 45 LpLhc superfamily proteins were identified, and their names were determined according to their orthologs in Arabidopsis and their chromosomal locations (**Table S12**). Phylogenetic analyses were performed for 34 AtLhc, 27 OsLhc and 45 LpLhc proteins to explore the phylogenetic relationship and evolutionary pattern. Our results showed that all 45 LpLhc proteins were grouped into four distinct families, including Lhc (including Lhca and Lhcb subfamily), Lil (including OHP, SEP, ELIP and Psb33 subfamily), PsbS and FCII, which is consistent with the classification of AtLhc proteins (**Figure 6A**; **Table S12**). Interestingly, the members of the Lhcb and ELIP subfamilies from the *L. perenne* genome were significantly expanded compared with those from Arabidopsis and rice (**Figure 6A**; **Table S12**).

To better understand the biological functions of *Lhc* genes in response to low-temperature stress, we then investigated the expression patterns of all 45 *LpLhcs* under low-temperature stress (**Figure 5B**; **Table S13**). The results showed that the expression levels of 20 (44.44%) *LpLhcs* were significantly altered in low temperature-stressed leaves for at least one time point, and 13 differentially expressed *LpLhcs* were coexpressed in all samples (**Figure 5C**). Notably, almost all members of the ELIP subfamily (92.86%, 13 out of 14 *LpELIPs*) had high expression levels in low temperature-stressed leaves and maintained high expression during all of the sampled low temperature-stressed time points (**Figure 5B**; **Table S13**). We further expanded the analysis of ELIP composition to seven sequenced Poaceae species to identify the expanded mechanism and assess the contribution of low-temperature adaptation for different species (**Table S14**). Our results showed that two *ELIPs* from maize are singletons, and all *ELIPs* in foxtail miller and sorghum are tandemly duplicated, while other studied Poaceae species have a mix of singleton and tandem gene copies (**Figure 6D**). Overall, most *ELIPs* in the studied Poaceae species (76.6%, 49 out of 64 *ELIPs*) were found in large tandem arrays (**Table S14**). In addition, Pooideae, having a highly successful low temperature-adapted lineage, tends to have more *ELIPs* than Panicoideae and Oryzoideae, and this phenomenon is more obvious in core Pooideae, suggesting that the high copy number of *ELIPs* may help combat rapid changes in light intensity and contribute to low temperature adaptation.

## 4 Discussion

Numerous studies have confirmed that Pooideae have successfully adapted to and diversified in cool climate ecosystems (Edwards and Smith, 2010; Vigeland et al., 2013). However, as a member of the core Pooideae species, the potential genetic mechanism underlying low-temperature adaptation in *L. perenne* is not well understood, and there is a lack of evidence at the genomic level. In this study, comprehensive comparative genomic and transcriptomic analyses were performed to illustrate the genomic basis of low-temperature adaptation in *L. perenne*. A total of 3,770 TDGs were identified in the *L. perenne* genome (**Table S1**), and chromosomes 4 and 5 contained the largest (728 TDGs, 19.31% of total TDGs) and lowest (396 TDGs, 10.50% of total TDGs) numbers of TDGs, respectively (**Figure 1A**; **Table S2**). Large-scale TDG cluster (more than five genes in the same cluster) analysis showed that 19 large-scale clusters (containing 99 TDGs) were identified, and 9 large-scale clusters were found to be associated with abiotic stress (**Table S4**). Among them, three clusters contain F-box genes, which encode proteins that play crucial roles in regulating various biological processes and abiotic stress responses by integrating almost all phytohormone signaling pathways (Yan et al., 2011; Jain et al., 2023). One cluster contains the AP2/ERF transcription factor, which has largely been implicated in abiotic stress responses by activating the expression of abiotic stress-responsive genes (Mizoi et al., 2012). In our study, 16 F-box and 5 AP2/ERF genes were identified resulting from tandem duplication events, suggesting that large-scale tandem clusters might participate in abiotic stress responses in *L. perenne* (**Table S4**). In addition, although the large-scale TDG clusters included uncharacterized proteins, some genes, including *V3.Lp_chr3_0G5746.1*, *V3.Lp_chr3_0G5750.1* and *V3.Lp_chr3_0G5754.1*, were induced by low temperature (**Table S10**), suggesting that these genes also contribute to low temperature resistance in *L. perenne* and could serve as potential novel genes related to low temperature resistance.

GO and KEGG enrichment analyses may provide valuable information for understanding the high-level functions and utilities of biological processes (Kanehisa et al., 2017). GO enrichment analysis showed that 3,770 *L. perenne* TDGs were enriched in 148 species-specific GO terms compared with *H. vulgare*, *B. distachyon*, *A. splendens* and *O. sativa* (**Table S6**). These unique GO terms included plant-type cell wall organization or biogenesis, inorganic anion transport and organic cation transport (**Figure 2C**; **Table S6**), suggesting that these GO terms participate in the modification of cell wall composition and promote intracellular ion homeostasis to avoid osmotic stress caused by abiotic stress (Ambroise et al., 2020; Xu et al., 2020a). In comparison with rice, 26 Pooideae-specific GO terms, including photoprotection and cellular response to light intensity, were identified (**Figure 2D**; **Table S7**), implying that Pooideae plants (such as *L. perenne*, *H. vulgare*, *B. distachyon* and *A. splendens*) can activate a number of highly dynamic photoprotective strategies depending on the light intensity under low temperature stress (Velitchkova et al., 2020). Photosynthesis-antenna proteins that play indispensable roles in the capture of solar energy as well as photoprotection under various stress conditions (Zhao et al., 2020), such as avoiding photooxidative damage in overwintering plants caused by low temperature (Oquist and Huner, 2003). Under chilling stress, 7.38% photosynthesis–antenna proteins were significantly induced in rice leaves (Li et al., 2022), and overexpression tomato LHC antenna protein gene (*LeLhcb2*) enhanced transgenic tobacco tolerance to chilling stress by alleviating photo-oxidation of PSII (Deng et al., 2014). Similarly, overexpression of *Rhododendron ELIP* in Arabidopsis conferred plant tolerance to freezing stress through rescuing photosystem (Liu et al., 2022a). The cytochrome P450 proteins (CYPs) participate in various metabolic pathways and play crucial roles in multiple processes, particularly roles in stress responses (Zeng et al., 2019). In *osmanthus fragrans*, 67 tandem duplicated *CYPs* were identified, and some of them were significantly induced by cold stress (Liu et al., 2022b). Integrating genomic and transcriptomic analyses revealed expanded cytochrome P450 contribute to stress adaptation for Pistachio (Zeng et al., 2019). In addition, tandem duplicated auxin response factor genes (*ARFs*) have been reported in Arabidopsis (Okushima et al., 2005) and peach (Shen et al., 2015), and the duplicated *Aux/IAA14* regulates microRNA-mediated cold stress response in Arabidopsis (Okushima et al., 2005; Aslam et al., 2020). In our study, photosynthesis-antenna proteins pathway was identified to be a unique KEGG pathway for *L. perenne*, and TDGs were also enriched in drug metabolism-cytochrome P450 and plant hormone signal transduction pathway (**Figure 3C**; **Table S8**), suggesting that TDGs might play important roles in the response of *L. perenne* to environmental stimuli, particularly roles in low-temperature responses.

The release transcriptional profiles provide a great opportunity to understand the expression patterns in different tissues and in response to stress responses (Farrell et al., 2014; Abeynayake et al., 2015). Duplication events, which occur frequently in most plants, contribute to species diversification and functional innovation and play crucial roles in plant adaptation to stressful habitats (Murat et al., 2010; Wang et al., 2021). In our study, the differential expression analysis revealed that 9.91% (6,582 out of 66,405) of genes were identified as DEGs in *L. perenne*, while a higher proportion (17.48%, 659 out of 3,770) of TDGs were significantly affected by low temperature (**Figure 5C**), suggesting that TDGs might play more important roles in contributing to low temperature tolerance for *L. perenne*. Among these 659 differentially expressed TDGs, 39.8% TDGs (262 out of 659) were shared at all time points under low-temperature stress (**Figure 5B**), implying that a considerable proportion of TDGs display conservative functions and retain stress responsiveness. A total of 12.4% (82 out 659), 11.4% (75 out of 659) and 8.5% (56 out of 659) differentially expressed TDGs were specifically expressed in 9, 13 and 17 d low temperature-stressed leaves, respectively (**Figure 5B**), suggesting that these TDGs in *L. perenne* underwent functional divergence in the process of evolution. Determining the number of synonymous substitutions per synonymous site (Ks) between paralogs allows us to trace the history of duplication events and detect the main duplication events that occurred in plants (Vanneste et al., 2015; Ren et al., 2018). Genomic synteny analysis for 2,042 gene pairs (3,770 TDGs) indicated that *L. perenne* might has undergone a duplication event approximately 7.69 Mya (Ks = 0.10) (**Figure 1B**), while the distribution of Ks for 262 shared differentially expressed TDGs at all time points under low-temperature stress revealed that these stress-responsive TDGs might result from duplication events that occurred approximately 20-31 Mya (Ks = 0.36) (**Figure 5D**). These results suggested that recent duplication events led to the expansion of TDGs, and stress-responsive TDGs mainly originated from earlier duplication events and played a more important role in facilitating the adaptation of *L. perenne* to low temperature.

Light-harvesting chlorophyll a/b-binding (LHC) proteins play indispensable roles in capturing solar energy during photosynthesis, photoprotection of photosystem II (PSII) and alleviation of oxidative stress caused by stress conditions (de Bianchi et al., 2008; Luo et al., 2022). To study their contributions to low temperature stress, we identified a total of 45 LpLhc superfamily proteins in the *L. perenne* genome (**Table S12**). Comparative analysis with other species found that the ELIP subfamily from *L. perenne* was significantly expanded (**Figure 6A**; **Table S12**). Increasing evidence has confirmed that ELIPs accumulate in photosynthetic tissue under various abiotic stresses, including cold, drought and heat (Adamska and Kloppstech, 1994; VanBuren et al., 2019), and play an important role in protecting against photooxidative damage by chlorophyll binding and stabilization of the photosynthetic complex (Hutin et al., 2003). For example, ELIP3 showed significant accumulation in *Chlamydomonas reinhardtii* under cold stress and helped survival of the cell under photooxidative stress, and the phenotype results of mutant and overexpression plants revealed that *ELIP3* plays an important role in protecting the photosystem under photooxidative stress at low temperatures by regulating the redox state of the cell (Lee et al., 2020). Overexpression of a *M. truncatula ELIP* in *N. benthamiana* enhanced the resistance to freezing, chilling and osmotic stress by protecting the chloroplast against photooxidative damage (Araújo et al., 2013). In the present study, 13 out of all 14 *LpELIPs* were significantly induced in leaves by low temperature (**Figures 6B, C**), suggesting that *LpELIPs* actively responded to low temperature. Expansion of *ELIPs* was previously reported in *B. hydrometrica* (Xiao et al., 2015), *S. lepidophylla* (VanBuren et al., 2018b) and *L. subracemosa* (VanBuren et al., 2018a). A comparative genomics analysis of 75 sequenced land plants showed that massive tandem proliferation of *ELIPs* supports convergent evolution of desiccation tolerance across land plants, and expression analysis revealed that *ELIPs* had low or undetectable expression under well-watered conditions but exhibited higher expression levels under dehydration stress and that expression increased throughout the progression of dehydration stress (VanBuren et al., 2019). In our study, the expression of all TDGs was significantly lower than that of all genes, and 14 *LpELIPs* had low or undetectable expression in all six tissues under normal conditions (**Figure 4B**; **Table S15**), while the expression level of *LpELIPs* rapidly increased and continuously maintained higher expression during all of the sampled stress time points (**Figure 6B**), implying that *ELIPs* might be involved in various stress responses and play an important role in regulating the low temperature tolerance of *L. perenne*. In addition, comparative genomics analysis revealed that most *ELIPs* (76.6%, 49 out of 64 ELIPs) resulted from tandem duplication events, and Pooideae has more *ELIPs* than Panicoideae and Oryzoideae, particularly in core-Pooideae species (**Figure 6D**; **Table S14**), suggesting that tandem duplication might increase the absolute transcript abundance of *ELIPs*, improve photoprotective capacity, and contribute to low temperature adaptation in *L. perenne*. Our results provide insights into the roles of tandem duplication in the evolution and low-temperature adaptation of *L. perenne* and provide candidate gene resources for molecular breeding in *L. perenne*, although the specific functionality of ELIPs needs further verification.

## 5 Conclusions

In this study, we examined the TDG signatures and analyzed their contributions to adaptive evolution in *L. perenne*, including *L. perenne*, which might has undergone a duplication event approximately 7.69 Mya, and TDGs might contribute to the environmental adaptability of *L. perenne*. By transcriptomic analysis, we also found that these TDGs had lower expression than all genes in all six different tissues, while a higher proportion of TDGs were significantly affected by low-temperature stress, and those stress-responsive TDGs mainly resulted from the duplication event that occurred approximately 20-31 Mya. In addition, *ELIPs* could rapidly respond and continuously maintain higher expression levels during all of the sampled stress time points, suggesting that the expanded *ELIPs*, which were mainly caused by tandem duplication events, participate in low temperature responses and help facilitate the adaptation to low temperature in *L. perenne*. Our results provide an important and valuable basis for understanding *L. perenne* adaptation to low-temperature stress and facilitate the genetic improvement of molecular breeding in *L. perenne*.

## Data availability statement

The original contributions presented in the study are included in the article/**Supplementary Material**. Further inquiries can be directed to the corresponding author.

## Author contributions

WW and YL conceived the experiments and wrote the manuscript, WW, YL, YH and MW performed the experiments and analyzed the data, XL and JW assisted in data analysis, SF and YY revised the manuscript. All authors contributed to the article and approved the submitted version.
